# Supplementary material for: Leiomyogenic Tumor of the Spine: A Systematic Review
Source: Cancers (Basel). 2024 Feb 10;16(4):748. doi: 10.3390/cancers16040748 (PMC10887395; doi:10.3390/cancers16040748)
Supplement: Supplementary file 1 [file cancers-16-00748-s001.zip › cancers-2816665-supplementary.pdf]

Table 1. Summary Data from Included Studies.

Author	Case Number	Year	Age	Gender	Tumor Location	Primary Treatment Modality	Adjuvant Therapy	Long Term Outcomes	Status [A/D]
**Kang et al**	1	2011	30	F	Thoracic Spine	Fixation and Resection	Resection and Biopsy	Clinically Stable	A
**Hekster et al**	2	1994	43	F	Cervical Spine	Decompression and Resection	Danazol	Clinically stable	A
**Shirzadi et al**	3	2012	84	F	Lumbar Spine	Laminectomy, Decompression, Resection, Fixation	Radiation	-	/
**Koda et al**	4	2018	72	M	Thoracic Spine	en bloc spondylectomy of T12	Radiation	Clinically Stable	A
**Ehresman et al**	5	2018	24	F	Thoracic Spine	Laminectomy, Fixation, and Resection	Radiation	-	/
**Silva et al**	6	2005	29	F	Thoracic and Lumbar Spine	Decompression and Resection	Radiation and Chemotherapy	Clinically Stable	/
**Yassaad et al**	7	2016	37	F	Thoracic and Lumbar Spine	Decompression, Fixation, and Resection	-	Recurrence in Skull	D
**Barber et al**	8	2019	49	F	Thoracic Spine	Laminectomy and Decompression	Depo Lupron, Arimidex, and Radiation	Clinically Stable	A

**Munjal et al**	9	2017	33	F	Thoracic and Lumbar Spine	Laminectomy and Resection	-	Clinically Stable	A
**Strong et al**	10	2015	52	F	Lumbar Spine	Laminectomy and Resection	Radiation and Chemotherapy	Clinically Stable	A
**Calafiore et al**	11	2020	62	M	Thoracic Spine	Decompression, Fixation, and Resection	-	Clinically Stable	A
**Yang et al**	12	2014	19	F	Thoracic Spine	Laminectomy, Resection	-	Clinically Stable	A
**Zong et al**	13	2018	51	F	Lumbar Spine	Corpectomy, Resection, and Vertebroplasty	Tamoxifen	Clinically Stable	A
**Ghani et al**	14	2021	70	F	Lumbar and Sacral Spine	-	Radiation	Receiving Palliative Care	A
**Patibandla et al**	15	2017	48	F	Cervical Spine	Decompression, Resection, and Fixation	-	Clinically Stable	A
**Yang et al**	16	2017	47	F	Thoracic Spine	Corpectomy, Fixation, Resection	-	Clinically Stable	A
**Ahmad et al**	17	2017	47	F	Thoracic Spine	Decompression, Fixation, and Resection	Radiation	Clinically Stable	A
**Dohi et al**	18	2003	67	F	Sacral Spine	Resection	Radiation	Clinical Deterioration	/

**Kim et al**	19	2019	64	F	Thoracic Spine	Laminectomy and Resection	Chemotherapy	Clinical Deterioration	D
**Alessi et al**	20	2003	43	F	Sacral Spine	Laminectomy and Resection	Chemotherapy	Clinically Stable	/
**Pluemvitayaporn et al**	21	2017	61	F	Thoracic Spine	Total en bloc spondylectomy	Chemotherapy	Clinically Stable	A
**Ramirez-Villaescusa et al**	22	2017	62	F	Thoracic Spine	Total en bloc spondylectomy	Radiation	Clinically Stable	A
**Iacoban et al**	23	2022	45	M	Thoracic Spine	Corpectomy, Laminectomy, Fixation, Resection	Radiation and Chemotherapy	-	/
**Vasudeva et al**	24	2017	62	M	Thoracic Spine	Resection	Radiation	Clinically Stable	A
**Agarwal et al**	25	2017	50	F	Thoracic Spine	Laminectomy Decompression, Fixation, and, Resection	Chemotherapy	Recurrence with subsequent Resection. Clinically Stable	A
	26	2017	45	M	Thoracic Spine	Corpectomy, Laminectomy, Fixation	-	Clinical Deterioration	D
	27	2017	40	F	Thoracic Spine	Laminectomy, Fixation, and Resection	Radiation	Clinically Stable	A
	28	2017	58	F	Thoracic Spine	Laminectomy, Fixation, Resection	Radiation	Clinically Deteriorated	D

	29	2017	48	M	Thoracic Spine	Osteotomy, Laminectomy, Resection	Radiation, Chemotherapy	Recurrence, Clinical Deterioration	D
**Inoue et al**	30	2020	58	F	Lumbar Spine	-	Letrozole	Clinically Stable	A
**Hur et al**	31	2015	42	F	Lumbar Spine	Paravertebral Muscle Dissection, Fixation, Resection	/	-	/
**Shimoda et al**	32	1998	61	F	Lumbar Spine	-	-	Advanced Metastatic Disease, Clinical Deterioration	D
**Takemori et al**	33	1993	47	F	Thoracic Spine	T8 vertebra replacement	Chemotherapy	Clinically Stable	A
**Aksoy et al**	34	2002	70	F	Thoracic Spine	Resection	Chemotherapy	/	/
**Arnesen et al**	35	1992	56	F	Thoracic Spine	Laminectomy, Resection	Radiation	Clinically Stable	A
**Nanassis et al**	36	1999	46	F	Thoracic Spine	Decompression, Resection	-	Metastatic Disease	A
**Jayakody et al**	37	2011	44	F	Thoracic Spine	Fixation, Resection	/	Receiving Palliative Care	A

**Lehman et al**	38	2007	45	M	Cervical Spine	Intracapsular Resection	Reoperation for Gross Total Resection, Radiation, and Chemotherapy	Clinically Stable	A
**Sun et al**	39	2022	29	M	Cervical Spine	Decompression, Corpectomy, Fixation, Resection	Radiation and Chemotherapy	Clinically Stable	/
**Sucu et al**	40	2011	25	F	Cervical Spine	Partial spondylectomy, Fixation and Resection	Radiation and Chemotherapy	Clinically Stable	A
**Ido et al**	41	2002	83	M	Thoracic Spine	Decompression and Fixation	/		/
**Ziewacz et al**	42	2012	25	M	Thoracic Spine	Laminectomy, Vertebrectomy, and Fixation	Radiation and Chemotherapy	Recurrence with Clinical Deterioration	D
	43	2012	61	F	Lumbar Spine	Laminectomy, Fixation, and Resection	Radiation and Chemotherapy	Clinically Stable	A
	44	2012	35	F	Thoracic Spine	Laminectomy, Fixation, and Resection	Radiation and Chemotherapy	Recurrence with Clinical Deterioration	D
	45	2012	57	F	Thoracic Spine	Laminectomy, Corpectomy, and Fixation	Radiation and Chemotherapy	Recurrence with Clinical Deterioration	D

	46	2012	57	F	Lumbar and Sacral Spine	Laminectomy and Resection	Radiation and Chemotherapy	Recurrence with Clinical Deterioration	D
	47	2012	51	F	Thoracic Spine	Laminectomy, Fixation, and Resection	-	Recurrence with Clinical Deterioration	D
	48	2012	55	M	Thoracic Spine	Laminectomy, Fixation, and Resection	Radiation and Chemotherapy	Recurrence with Clinical Deterioration	D
	49	2012	66	M	Thoracic Spine	Laminectomy and Resection	Chemotherapy	Clinical Deterioration	D
**Tahara et al**	50	2016	61	F	Thoracic	Resection, Arterial Embolization	Radiation	Clinically Stable	A
**Ganau et al**	51	2002	23	F	Lumbar and Sacral Spine	Arterial Embolization, Resection	Radiation	/	/
**Elhammady et al**	52	2007	45	F	Lumbar Spine	Corpectomy, Resection, Fixation	Radiation and Chemotherapy	Tumor Recurrence, Clinically Stable	A
	53	2007	46	F	Thoracic Spine	Laminectomy, Fixation	-	Clinically Stable	A
	54	2007	36	F	Lumbar Spine	Laminectomy, Fixation	Chemotherapy	Clinical Deterioration 9 years after index operation	D
	55	2007	42	F	Lumbar Spine	Laminectomy, Fixation	-	Clinically Stable	A

	56	2007	47	F	Thoracic and Lumbar Spine	Corpectomy, Decompression, Fixation	Chemotherapy	Clinical Deterioration due to Metastatic disease 13 years after index operation	D
**Kato et al**	57	2020	52	F	Thoracic Spine	Total en bloc spondylectomy	-	Tumor Recurrence, Clinical Deterioration	D
	58	2020	59	F	Thoracic and Lumbar Spine	Total en bloc spondylectomy	-	Clinical Deterioration	D
	59	2020	49	M	Thoracic Spine	Total en bloc spondylectomy	Chemotherapy	Clinical Deterioration	D
	60	2020	56	M	Lumbar Spine	Total en bloc spondylectomy	-	Clinical Deterioration	D
	61	2020	62	F	Thoracic Spine	Total en bloc spondylectomy	Chemotherapy	Clinical Deterioration	D
	62	2020	69	M	Thoracic Spine	Total en bloc spondylectomy	Chemotherapy	Clinical Deterioration	D
	63	2020	24	F	Thoracic Spine	Total en bloc spondylectomy	Chemotherapy	Clinical Deterioration	D
	64	2020	57	M	Thoracic Spine	Total en bloc spondylectomy	Chemotherapy	Clinically Stable	A

	65	2020	56	M	Thoracic Spine	Total en bloc spondylectomy	-	Clinical Deterioration	D
	66	2020	44	F	Lumbar Spine	Total en bloc spondylectomy	Chemotherapy and Radiation	Tumor Recurrence, Clinically Stable	A
**Xiao et al**	67	2019	61	F	Lumbar Spine	Resection, Fixation	Bisphosphonate	Clinically Stable	A
	68	2019	49	F	Thoracic Spine	Resection, Fixation	Chemotherapy	Clinical Deterioration	D
	69	2019	56	M	Lumbar and Sacral Spine	Resection, Fixation	Chemotherapy	Clinically Stable	A
	70	2019	22	F	Cervical and Thoracic Spine	Resection, Fixation	-	Clinically Stable	A
	71	2019	34	M	Lumbar Spine	Resection, Fixation	-	Clinically Stable	A
	72	2019	44	F	Lumbar Spine	Resection, Fixation	-	Clinically Stable	A
	73	2019	49	F	Cervical Spine	Resection, Fixation	Chemotherapy and Bisphosphonates	Clinically Stable	A
	74	2019	55	F	Lumbar Spine	Resection, Fixation	-	Clinically Stable	A
	75	2019	55	F	Lumbar Spine	Resection, Fixation	Bisphosphonates	Clinically Stable	A
	76	2019	51	F	Sacral Spine	Resection, Fixation	Radiation	Clinically Stable	A

	77	2019	47	F	Lumbar Spine	Resection, Fixation	-	Clinically Stable	A
	78	2019	73	M	Thoracic Spine	Resection, Fixation	Chemotherapy and Bisphosphonates	Clinically Stable	A
**Elmaci et al**	79	2021	34	F	-	-	Radiation and Chemotherapy	Clinically Stable	A
**Tan et al**	80	2013	44	F	Cervical and Thoracic Spine	Laminoplasty with Resection	Radiation and Chemotherapy	/	/
**Kawashima et al**	81	2020	61	M	Thoracic Spine	Resection and Fixation	Radiation and Chemotherapy	Clinical Deterioration	D
	82	2020	47	M	Thoracic Spine	Laminectomy, Resection, and Fixation	Radiation and Chemotherapy	Clinical Deterioration	D
	83	2020	48	M	-	-	Radiation and Chemotherapy	Metastatic Disease, Clinical Deterioration	D

Table S2: Analysis of Surgical methods

	Average follow up time (months)	Resolution of symptoms (Yes, No, No Data)	Survival Status (Alive, Dead, No Data)
Total En-bloc Spondylectomy	43	1 (8.3%),0(0.00%),11 (91.7%)	4 (33.3%),8(67.3%),0 (0.0%)
Other	14	19 (26.8%),9(12.7%),43(60.6%)	40 (56.3%),19(26.8%),12(16.9%)
